# A Very Rare Case of Hypereosinophilic Syndrome Secondary to Natural Killer/T-Cell Lymphoma

**DOI:** 10.1155/2018/5965029

**Published:** 2018-03-26

**Authors:** Takanori Yamamoto, Atsushi Kamijo, Tadao Nakazawa, Kei Nakajima, Keita Kirito, Norio Komatsu, Keisuke Masuyama

**Affiliations:** ^1^Department of Otorhinolaryngology and Head & Neck Surgery, University of Yamanashi, Yamanashi, Japan; ^2^Department of Otorhinolaryngology and Allergy Center, Saitama Medical University, Saitama, Japan; ^3^Department of Pathology, University of Yamanashi, Yamanashi, Japan; ^4^Department of Hematology and Oncology, University of Yamanashi, Yamanashi, Japan; ^5^Department of Hematology, Juntendo University School of Medicine, Tokyo, Japan

## Abstract

Hypereosinophilic syndrome (HES) is a systemic disease characterized by an increased peripheral blood eosinophil count accompanied by systemic organ dysfunction. HES is classified into idiopathic HES, primary (neoplastic) HES (HES_N_), and secondary (reactive) HES (HES_R_). In this case report, a patient who developed peripheral blood eosinophilia and granulation tissue in the pharynx and paranasal sinus, which was initially diagnosed as chronic eosinophilic leukemia (CEL), categorized as HES_N_, but was eventually identified after the patient had died as natural killer/T-cell (NK/T) lymphoma, nasal type (ENKL), categorized as HES_R_, is presented. ENKL-induced HES is very rare but must be considered.

## 1. Introduction

Hypereosinophilic syndrome (HES), organ dysfunction due to eosinophilia, has various etiologies. In this case report, a patient diagnosed as having chronic eosinophilic leukemia (CEL) because FIP1-like-1-platelet-derived growth factor receptor *α* (*FIP1L1-PDGFRA*: FP) chimeric gene was initially detected by reverse transcription polymerase chain reaction (RT-PCR) expression analysis is described. However, the patient was refractory to treatment and subsequently died, after which the diagnosis was revised to nasal-type NK/T-cell lymphoma. NK/T-cell lymphoma is rarely the cause of HES and can be difficult to diagnose. It is therefore essential to consider the possibility of this disease to make a correct diagnosis.

## 2. Case Report

The patient was a 23-year-old woman who was referred to our hospital in mid-June of XXXX (day 1) with a chief complaint of nasal congestion. Before visiting our hospital, the patient had been diagnosed by her local otolaryngologist with rhinosinusitis and was taking oral antimicrobial drugs, but her condition did not improve, and she developed a fever (38°C) at the beginning of June. The patient had a previous history of systemic lupus erythematosus (SLE) for which she had undergone treatment from the age of 15 years. However, the SLE was well controlled with 5 mg/day of oral prednisolone.

**Figure 1:**
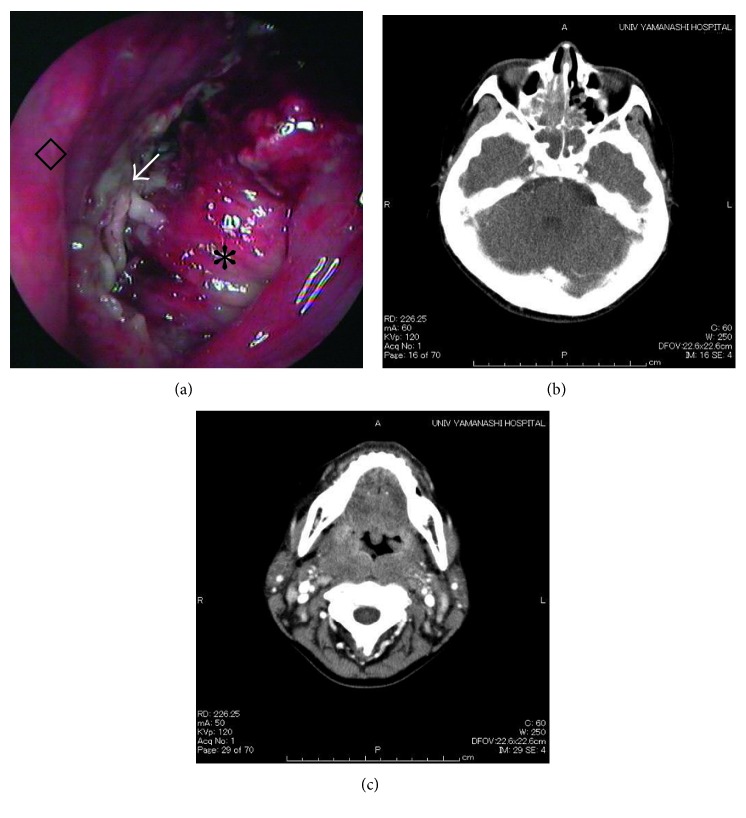
Scope image and computed tomography (CT) findings. (a) Scope image of the left nasal cavity. White granulation tissue is observed (arrow). ^∗^: inferior turbinate and ◇: nasal septum. (b) Axial CT with contrast demonstrating soft tissue lesions or effusions in the ethmoid and sphenoid sinuses. (c) Axial CT with contrast demonstrating mucosal and tissue swelling in the pharynx.

Nasal fiberscopy findings showed that the nasal cavity and nasopharynx were filled with a viscous exudate and granulation tissue (Figure 1(a)). Computed tomography (CT) scans demonstrated space-occupying lesions in the bilateral ethmoid and sphenoid sinuses and a soft tissue lesion from the nasopharynx to the oropharynx (Figures 1(b) and 1(c)). Hematology findings showed a C-reactive protein (CRP) count of 7.32 mg/dl and a peripheral blood white blood cell (WBC) count of 14,000/*μ*l, consisting of a neutrophil count of 9,660/*μ*l (69%) and eosinophil count of 2,940/*μ*l (21%), indicating eosinophilia without blasts. Lactate dehydrogenase (LDH) was slightly elevated at 313/IU, and soluble interleukin-2 receptor (sIL-2R) was also high at 2200 U/ml.

Infection was initially suspected, but antimicrobial treatment was ineffective. The results of subsequent biopsies of tissue from the nasal cavity and pharynx led to a diagnosis of granulation tissue with significant eosinophil infiltration, for which steroid pulse therapy was given. The patient's eosinophil count decreased transiently, but it then increased again, so bone marrow aspiration was performed. The results did not indicate any blasts in the bone marrow; they only showed that mature eosinophils comprised half of the infiltrating cells. The results of Giemsa- (G-) banded chromosome analysis performed at that time did not show any abnormalities. Abdominal ultrasound showed hepatosplenomegaly. However, RT-PCR expression analysis was positive for the *FIP1L1-PDGFRA* chimeric gene. The patient was therefore diagnosed with chronic eosinophilic leukemia (CEL). From day 60, the patient was started on oral therapy with the tyrosine kinase inhibitor imatinib (100 mg/day), after which her eosinophil count decreased from 21,366/*μ*l to 12,079/*μ*l the next day. However, the swelling in the patient's larynx and neck worsened, so the imatinib dose was increased to 400 mg/day. The eosinophil count decreased transiently again, but it increased on day 65 to 27,424/*μ*l. On day 75, the imatinib was switched to oral hydroxyurea (1500 mg/day), but it was ineffective, and she died on day 85.

**Figure 2:**
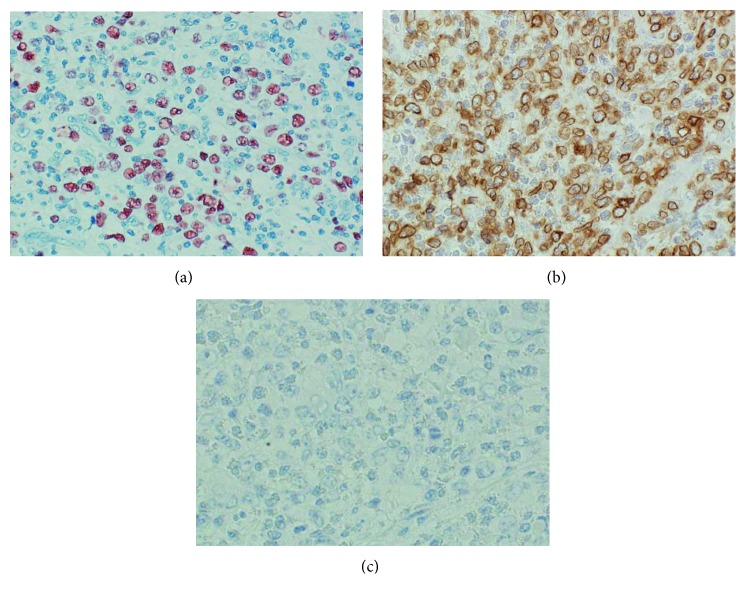
Morphological and immunohistochemical characteristics of the lymphoma (×200). (a) In situ hybridization with EBER showing strong Epstein–Barr virus expression by a large atypical lymphocyte. (b) Immunohistochemical staining with CD3 showing strong positive staining. (c) Immunohistochemical staining with CD56 showing negative staining.

The fact that the patient did not improve on imatinib therapy despite testing positive for the *FIP1L1-PDGFRA* fusion gene led to testing for the imatinib-resistant PDGFRA-T674I mutant, but the results were negative. Retesting for the FP chimeric gene was then requested at another facility after the patient died, but all test results were negative. Reanalysis of pathology specimens yielded positive results for Epstein–Barr virus-encoded early small RNA (EBER), cluster of differentiation 3 (CD3), CD4, and granzyme A, and negative results for CD56 (Figures 2(a)–2(c)), leading to an eventual diagnosis of nasal-type extranodal NK/T-cell lymphoma (ENKL).

## 3. Discussion

A case of nasal-type ENKL with concomitant HES was described. Since the initial PCR detected the *FIP1L1-PDGFRA* chimeric gene and a pathological diagnosis of NK/T-cell lymphoma was not made, there was a delay in providing appropriate treatment, which led to an unfortunate outcome.

The Working Conference on Eosinophilic Disorders and Syndromes proposed a classification of HES in 2011 [1]. This classification defines hypereosinophilia (HE) as a peripheral blood eosinophil count ≥1500/*μ*l recorded on at least two occasions with a minimum interval of four weeks, or as extensive tissue infiltration by eosinophils. Depending on the cause, HE is classified as (1) familial variant HE, HE_FA_; (2) HE of undetermined significance (no clear underlying cause and no clinical symptoms), HE_US_; (3) primary (clonal/neoplastic), HE_N_; and (4) secondary (associated with lymphoma, etc.), HE_R_. HE accompanied by organ dysfunction due to eosinophilia is defined as HES, which is classified as either idiopathic HES, primary (neoplastic) HES (HES_N_), or secondary (reactive) HES (HES_R_). Although this patient was seen in 2007 before this classification was proposed, if this classification was to be applied retrospectively, then the patient would have initially been diagnosed with HES_N_ and finally with HES_R_. According to the 2016 World Health Organization (WHO) classification [2], eosinophilic leukemia with no existing gene mutation, with blasts in the bone marrow or blood, and with clonal eosinophilia is classified as “chronic eosinophilic leukemia, not otherwise specified” (CEL, NOS). Separate from this classification, pathological conditions based on four molecular abnormalities, namely, PDGFRA, PDGFRbeta (PDGFRB), fibroblast growth factor receptor 1 (FGFR1), and *PCM1-JAK2* (fusion gene resulting from a t(8;9)(p22;p24) chromosomal translocation), are classified as myeloid/lymphoid tumors, all of which belong to the HES_N_ variant.

In 2003, Cools et al. identified the FP chimeric gene in nine of 16 HES patients [3]. Patients who carry this fusion gene are eligible for treatment with the tyrosine kinase inhibitor imatinib, which has been reported to be very effective [3, 4]. However, treatment with imatinib proved to be ineffective in this patient. Imatinib tolerance in individuals with the FP fusion gene is often caused by the T674I mutation occurring in the adenosine triphosphate- (ATP-) binding region, but it is also known to occur in those with S601P/L629P and D842V kinase domain mutations. Examination for the T674I mutation was performed in the present patient, but it was not detected. FP fusion gene searches typically use RT-PCR analysis, and this method was also used in reaching the initial diagnosis of eosinophilia due to myeloproliferative neoplasms involving the *FIP1L1-PDGFRA* chimeric gene. However, Sada et al. [5] reported that when performing RT-PCR with commonly used primer sets, an artificial splicing mechanism in the PCR process causes the FP chimeric gene to form even in the gene samples of healthy individuals. Diagnosis combining the use of fluorescence in situ hybridization (FISH) technique encompassing the three regions of the 4q12 cleavage site is therefore currently recommended.

After the patient's death, the cause of the eosinophilia was attributed to nasal-type extranodal NK/T-cell lymphoma (ENKL). All types of lymphomas are reportedly capable of triggering eosinophilia [6], but nasal-type NK/T-cell lymphoma is very rare in HES patients. The only case, a 21-year Bolivian woman with hemophagocytic syndrome-associated NK/T-cell lymphoma, was reported in English literature [7]. In addition to ENKL, the lymphocyte variant HE is also reported to accompany chronic active Epstein–Barr virus (CAEBV) infection, with EBV-infected TV beta 5.1 T-cell clones identified as causing the overproduction of Th2 cytokines [8]. The involvement of EBV has also been demonstrated in the onset of NK/T-cell lymphoma, so eosinophilia is believed to occur secondary to the abnormal secretion of cytokines induced by EBV-infected NK/T tumor cells.

Most cases of ENKL are NK cell-derived lymphomas expressing the CD3− CD56+ phenotype without T-cell receptor gene rearrangement. The present patient expressed the CD3+ CD56− phenotype, which appears to be relatively rare in ENKL. Wang et al. [9] reported that approximately 20% of 288 early-stage ENKL patients were categorized as CD56−. They also found that the complete remission rate was significantly lower in CD56− ENKL than in CD56+ ENKL (60.8% versus 80.6%) and that the overall survival was significantly lower in CD56− ENKL, stating that CD56 expression is an independent predictor of survival.

In addition, Li et al. compared nasal ENKL and ENKL of the extranasal upper aerodigestive tract and found that CD56-negative lymphomas are more common in the latter (16.7% and 53.6%, resp.) [10].

ENKL lesions consist mainly of necrotic tissue, which often complicates pathological diagnosis and can necessitate several biopsies. Although rare, ENKL must also be considered as a potential etiology of HES.

## 4. Conclusion

A patient who developed peripheral blood eosinophilia and granulation tissue in the pharynx and paranasal sinus, which was initially diagnosed as CEL, but which was eventually identified as ENKL after the patient had died, was presented. Since various diseases present with peripheral blood eosinophilia, correct differential diagnosis, though challenging, followed by appropriate treatment is essential.
